# Cannabidiol attenuates alcohol-induced liver steatosis, metabolic dysregulation, inflammation and neutrophil-mediated injury

**DOI:** 10.1038/s41598-017-10924-8

**Published:** 2017-09-21

**Authors:** Yuping Wang, Partha Mukhopadhyay, Zongxian Cao, Hua Wang, Dechun Feng, György Haskó, Raphael Mechoulam, Bin Gao, Pal Pacher

**Affiliations:** 1 Laboratory of Cardiovascular Physiology and Tissue Injury, National Institute on Alcohol Abuse and Alcoholism, National Institutes of Health, Bethesda, MD USA; 2 Department of Clinical Microbiology and Immunology, Affiliated Hospital of Guiyang Medical University, Guiyang, Guizhou Province China; 3 Laboratory of Liver Diseases, National Institute on Alcohol Abuse and Alcoholism, National Institutes of Health, Bethesda, MD USA; 4 Department of Surgery and Center for Immunity and Inflammation, Rutgers New Jersey Medical School, Newark, New Jersey USA; 5 Institute for Drug Research, Medical Faculty, Hebrew University, Jerusalem, 91120 Israel

## Abstract

Cannabidiol (CBD) is a non-psychoactive component of marijuana, which has anti-inflammatory effects. It has also been approved by FDA for various orphan diseases for exploratory trials. Herein, we investigated the effects of CBD on liver injury induced by chronic plus binge alcohol feeding in mice. CBD or vehicle was administered daily throughout the alcohol feeding study. At the conclusion of the feeding protocol, serums samples, livers or isolated neutrophils were utilized for molecular biology, biochemistry and pathology analysis. CBD significantly attenuated the alcohol feeding-induced serum transaminase elevations, hepatic inflammation (mRNA expressions of TNFα, MCP1, IL1β, MIP2 and E-Selectin, and neutrophil accumulation), oxidative/nitrative stress (lipid peroxidation, 3-nitrotyrosine formation, and expression of reactive oxygen species generating enzyme NOX2). CBD treatment also attenuated the respiratory burst of neutrophils isolated from chronic plus binge alcohol fed mice or from human blood, and decreased the alcohol-induced increased liver triglyceride and fat droplet accumulation. Furthermore, CBD improved alcohol-induced hepatic metabolic dysregulation and steatosis by restoring changes in hepatic mRNA or protein expression of ACC-1, FASN, PPARα, MCAD, ADIPOR-1, and mCPT-1. Thus, CBD may have therapeutic potential in the treatment of alcoholic liver diseases associated with inflammation, oxidative stress and steatosis, which deserves exploration in human trials.

## Introduction

Chronic alcohol consumption is a leading cause of alcoholic liver disease in the USA and worldwide, which eventually may progress to cirrhosis or hepatocellular carcinoma in susceptible subjects^1,2^. Interestingly, while most heavy drinkers develop fatty liver, only about 20% of them will develop liver cirrhosis^3^. Although numerous advances have been made in the understanding the complex mechanisms of alcohol-induced steatohepatitis (involving inflammation and oxidative stress) in cellular systems and animal models, the translation of these findings to clinical practice is still limited.

Cannabidiol (CBD) is the most abundant non-psychoactive constituent of marijuana plant (Cannabis Sativa) with excellent safety profile in humans even after chronic use^4–6^. An extract containing 50% CBD (Sativex) is used for treatment of pain and spasticity associated multiple sclerosis in numerous European counties and Canada^6^. Recently, CBD received U.S. Food and Drug Administration (FDA) approval for the evaluation of its effect in refractory childhood epilepsy (Lennox-Gastaut Syndrome and Dravet Syndrome) and glioblastoma multiforme in phase 3 and 1 clinical trials, respectively. In multiple preclinical disease models, including cardiomyopathies^7–9^, nephrotoxicity^10^, neuroinflammation^6,11^, colitis^12^, diabetic complications^13^ and cancer^14^ CBD has been reported to exert potent anti-inflammatory and antioxidant effects^6^.

CBD improved brain and liver function in a fulminant hepatic failure-induced model of hepatic encephalopathy in mice^15^, decreased hepatic ischemia-reperfusion induced injury both in mice^16^ and rats^17^, attenuated alcohol-binge-induced injury in mice^18^ and hepatotoxicity of cadmium^19^ and cocaine^20^. Some of these studies have not explored the detailed mechanisms behind the protective effects of CBD against liver injury, but others proposed attenuation of the pro-inflammatory response and signaling (e.g. neutrophil infiltration, TNF-α, macrophage inflammatory protein-1α/2, cyclooxygenase 2, nuclear factor kappa B (NF-κB), oxidative/nitrative stress, stress signaling (p38MAPK and JNK) and cell death (apoptotic/necrotic), as well as promotion of autophagy^16–18^. CBD also attenuated bacterial endotoxin-triggered NF-κB activation and TNF-α production in isolated Kupffer cells^16^, and attenuated the intracellular adhesion molecule 1 expression in TNF-α stimulated primary human liver sinusoidal endothelial cells, and attachment of human neutrophils to the activated endothelium^16^. Silvestri *et al*. investigated the effects of CBD on lipid levels using *in vitro* and *in vivo* models of hepatosteatosis^21^. Using nuclear magnetic resonance-based metabolomics they demonstrated that CBD directly reduced lipid accumulation *in vitro* in hepatocytes and adipocytes and induced post-translational changes in CREB, PRAS40, AMPKa2 and several STATs indicating increased lipid metabolism^21^. They also demonstrated that CBD increased lipid mobilization and inhibited development of hepatosteatosis in zebrafish and obese mouse models^21^.

Since hepatic steatosis and neutrophil infiltration^1,22,23^ are critical pathological features of alcohol-induced liver injury, and the previously discussed studies suggested that CBD had beneficial effect on these processes, we investigated the effects of CBD on alcohol-induced liver steatosis, metabolic changes, inflammation and neutrophil-mediated oxidative injury, using a well-established model of chronic ethanol feeding plus binge alcohol gavage (NIAAA model)^24,25^, which closely relates to human drinking behavior^26^. Our results may have important implication for treatment of liver steatosis in alcoholic liver disease.

## Method

### Mice, alcohol feeding and treatments

Mice (C57BL/6 J) were purchased from The Jackson Laboratory (Bar Harbor, ME). All animal experiments were approved by the National Institute on Alcohol Abuse and Alcoholism Animal Care and Use Committee. The study was carried out in line with the National Institutes of Health (NIH) Guidelines for the Care and Use of Laboratory Animals. Ten to twelve-week-old female mice with weight over 25 g were subjected to the following feeding protocol. Initially mice were fed the control Lieber- DeCarli diet (Bio-Serv, Frenchtown, NJ) ad libitum for 5 days to acclimatize them to a liquid diet. Then mice were pair-fed with an isocaloric control diet (control-fed groups) or Lieber-DeCarli diet (alcohol-fed groups) containing 5% ethanol for 10 days. On day 11, ethanol and pair-fed mice were gavaged early morning with a single dose of ethanol (5 g/kg b.w.) or isocaloric dextrin-maltose, respectively, and sacrificed 9 hours later^24^.

CBD was isolated as described^27^. It was dissolved in vehicle solution (one drop of Tween 80 in 3 ml 2.5% dimethyl sulfoxide (DMSO) in saline) and injected i.p. (5 or 10 mg/kg/day) for 11 days during the ethanol exposure. Vehicle solution was used in control experiments.

### Biochemical assays

Serum alanine aminotransferase (ALT) and aspartate aminotransferase (AST) levels were determined using a clinical chemistry analyzer Idexx VetTest 8008 (Idexx Laboratories, Westbrook, ME, USA) as described^28^. Liver triglycerides were extracted with a 2:1 chloroform:methanol mixture and measured using the EnzyChrom Triglyceride Assay Kit (BioAssays Systems, Hayward, CA)^29^.

### Hepatic protein 3-nitrotyrosine (NT) content

Hepatic NT levels were determined using ELISA kit from Hycult biotechnology, Cell sciences, Canton, MA, USA, as described earlier^29^.

### Hepatic 4-hydroxynonenal (HNE) content

Levels of hepatic HNE were measured by using the kit from Cell Biolabs, San Diego, CA, USA. In brief, BS A or hepatic tissue homogenates (10 μg/ml) were absorbed on to the 96- well plates for 12 h at 4 °C. The HNE adducts contained in the samples were captured with anti-HNE antibody, followed by a HRP-conjugated secondary antibody. The HNE protein adducts in liver samples was determined based on standard curve generated with BSA-HNE according to the protocol supplied by the manufacturer^29^.

### Liver histology and immunohistochemistry

Liver specimens were fixed in 10% buffered formalin, embedded in paraffin, and cut into 5 µm sections. Paraffin embedded tissues were deparaffinized by changes of xylene and rehydrated in decreasing concentrations of ethanol. The sections were then subjected to hematoxylin and eosin (H&E) staining. For myeloperoxidase (MPO) staining slides were deparaffinized and hydrated in descending gradations of ethanol, followed by antigen retrieval procedure. Next, sections were incubated in 0.3% H_2_O_2_ in PBS to block endogenous peroxidase activity. The sections were then incubated with anti-MPO (Biocare Medical, Concord, CA, USA) or anti-malondialdehyde or anti-HNE (Genox, Baltimore, MD, USA) or anti –NT(cayman chemical, Ca) antibodies overnight at 4 °C in a moist chamber. Biotinylated secondary antibodies and ABC reagent were added according to the kit’s instructions (Vector Laboratories, Burlingame, CA, USA). Color development was induced by incubation with a DAB kit (Vector Laboratories) for 2–6 min, and the sections were counterstained^16,30^. Finally, the sections were dehydrated in ethanol and cleared in xylene and mounted. The specific staining was visualized and images were acquired using an IX-81 microscope (Olympus, Center Valley, PA). The morphometric examination was performed in a blinded manner by two independent investigators. The quantification of MPO positive cells was performed as described earlier^29^.

### Oil-O-Red staining and liver triglyceride content measurement

Liver samples embedded in optimal cutting temperature compound were cut at 10 μM sections and stained with Oil Red O to evaluate the hepatic lipid content. Briefly, cryosections were air-dried and fixed in 10% formalin and then stained with 0.5% Oil Red O in propylene glycol for 10 mins at 60 °C and subsequently washed with 85% propylene glycol. Sections were counterstained with hematoxylin, washed in water and mounted with aqueous solution.

Triglyceride content was measured from liver tissue by Triglyceride Quantification Colorimetric Kit (Biovision) according to manufacturer’s instruction.

### Isolation of hepatic leukocytes and flow cytometry analysis

Liver tissues were passed through a 70 µm cell strainer in phosphate-buffered saline (PBS), and the cell suspension was centrifuged at 30 *g* for 5 minutes to pellet the hepatocytes. The supernatant, which was enriched in nonparenchymal cells, was centrifuged at 300 *g* for 10 minutes. The pellet was resuspended in 15 ml of 35% Percoll (GE Healthcare, Pittsburgh, PA) and centrifuged at 500 *g* for 15 minutes. The resulting leukocyte pellet was resuspended in 2 ml of ACK lysing buffer (BioWhittaker, Walkersville, MD). After incubation for 5 minutes on ice, the cells were washed in PBS containing 2% fetal bovine serum. The cells were pre-incubated with Mouse BD Fc Block™ (purified rat anti-mouse CD16/CD32, clone 2.4G2, BD Biosciences, San Diego, CA) for 10 minutes at 4 °C and then stained with the designated antibodies for 30 minutes at 4 °C. The following antibodies were used: anti-F4/80 (clone BM8, eBioscience, San Diego, CA), anti-Gr-1 (clone RB6–8C5, eBioscience), anti-CD11b (clone M1/70, BD Biosciences), and anti-CD62L (clone MEL-14, BD Biosciences). Flow cytometry analysis was performed using a FACSCalibur (BD Biosciences).

### Oxidative burst assay from isolated liver leukocytes and human neutrophils

For detection of superoxide production we used Dihydrorhodamine 123 (30 mM stock in DMSO; Sigma Chemicals, St Louis, MO) at a final concentration of 100 μM with a modified method of Rothe *et al*.^31^. Isolated leukocytes from mouse liver were stained with Gr1-PE-Cy7.7 and CD11b-APC. Human granulocytes were isolated from human blood obtained from NIH clinical center by Polymorphprep according to manufacturer’s instruction (Axis-Shield, Norway). Human neutrophils were stained with CD11b-PE-Cy7.7 and CD66b-APC. The IRB was approved (99-CC-0168) by Department of Transfusion Medicine (NIH clinical center) for Collection and Distribution of Blood Components from Healthy Donors for *In Vitro* Research Use.

Cells were incubated with DHR and catalase (1000 U/ml, Sigma Chemicals, St Louis, MO) in the dark at 37 °C for 20 minutes. Cells loaded with DHR were treated with phorbol ester (PMA) at indicated concentration for 30 mins. For human cells, CBD and cannabinoid 2 receptor antagonist SR144528 were added as described in the text for 1 hour at 37 °C followed by phorbol ester (PMA) at 100 µg/ml for 30 mins. Finally, DHR intensities were measured by flow cytometry (FACS Calibur, BD Bioscience) in FL1 channel. Polymorphonuclear neutrophils were gated on the basis of their surface staining.

### Real-time quantitative polymerase chain reaction (real-time PCR)

Total RNA was extracted from liver tissue using the TRIzol reagent (Invitrogen, Carlsbad, CA) and treated with Turbo DNA-free (Ambion, Austin, TX) according to the manufacturer’s protocols. cDNA was synthesized using HT Fisrt stand CDNA synthesis kit (Qiagen, MD). Real-time PCR was performed in duplicate for each sample using the ABI PRISM 7500 Real-Time PCR System and SYBR Green Master Mix (Applied Biosystems) according to the manufacturer’s instructions. The specificity of transcript amplification was confirmed by melting curve profiles generated at the end of the PCR program. The expression levels of target genes were normalized to the expression of beta-actin and calculated based on the comparative cycle threshold Ct method (2^−ΔΔCt^). Primer sequences used were previously described^29,32^. The data shown represents the means ± SEM.

### Western Blot

Western blot was performed as described earlier^29^. The primary antibodies used are gp91phox (Biolegend, San Diego, CA), FASN, ACC1, PPAR-αl, and beta-actin (Abcam, Cambridge, MA).

### Quantitative ELISA

E–selectin and TNF-α were quantified using Simple Step Mouse E-Selectin ELISA Kit (Abcam) and Mouse TNF-alpha Quantikine ELISA Kit (R&D system) according to manufacturer’s instruction.

### Statistical Analysis

All the values are represented as mean ± SEM. Student’s t-test was used to determine difference between two groups. One-way analysis of variance (ANOVA) test was used to determine difference among more than two groups. Tukey’s post hoc tests were then performed to find significant differences between groups. The analysis was conducted using GraphPad-Prism 4 software. P < 0.05 was considered statistically significant.

## Results

### CBD treatment attenuates chronic-plus-binge ethanol-induced liver injury and steatosis

**Figure 1:**
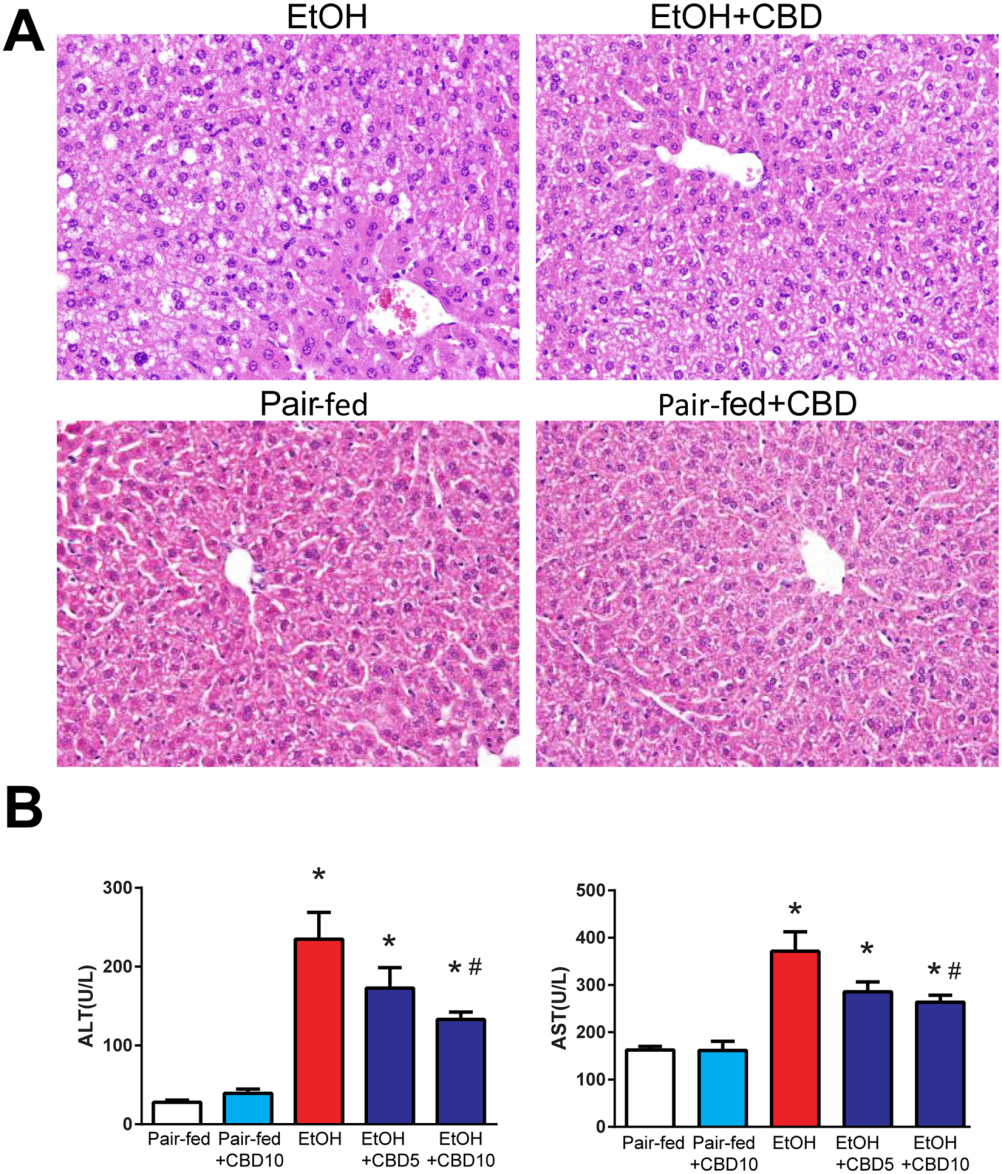
Effect of cannabidiol treatment on chronic-binge ethanol-induced liver injury. (**A**) Representative H&E staining of liver sections (**B**) and serum ALT, AST levels in indicated groups. Values represent means ± SEM (n = 4–7). *P < 0.05 vs. Pair-fed group, ^#^ *P* < 0.05 vs. EtOH group determined by One-way ANOVA, followed by Tukey’s post-hoc test.

**Figure 2:**
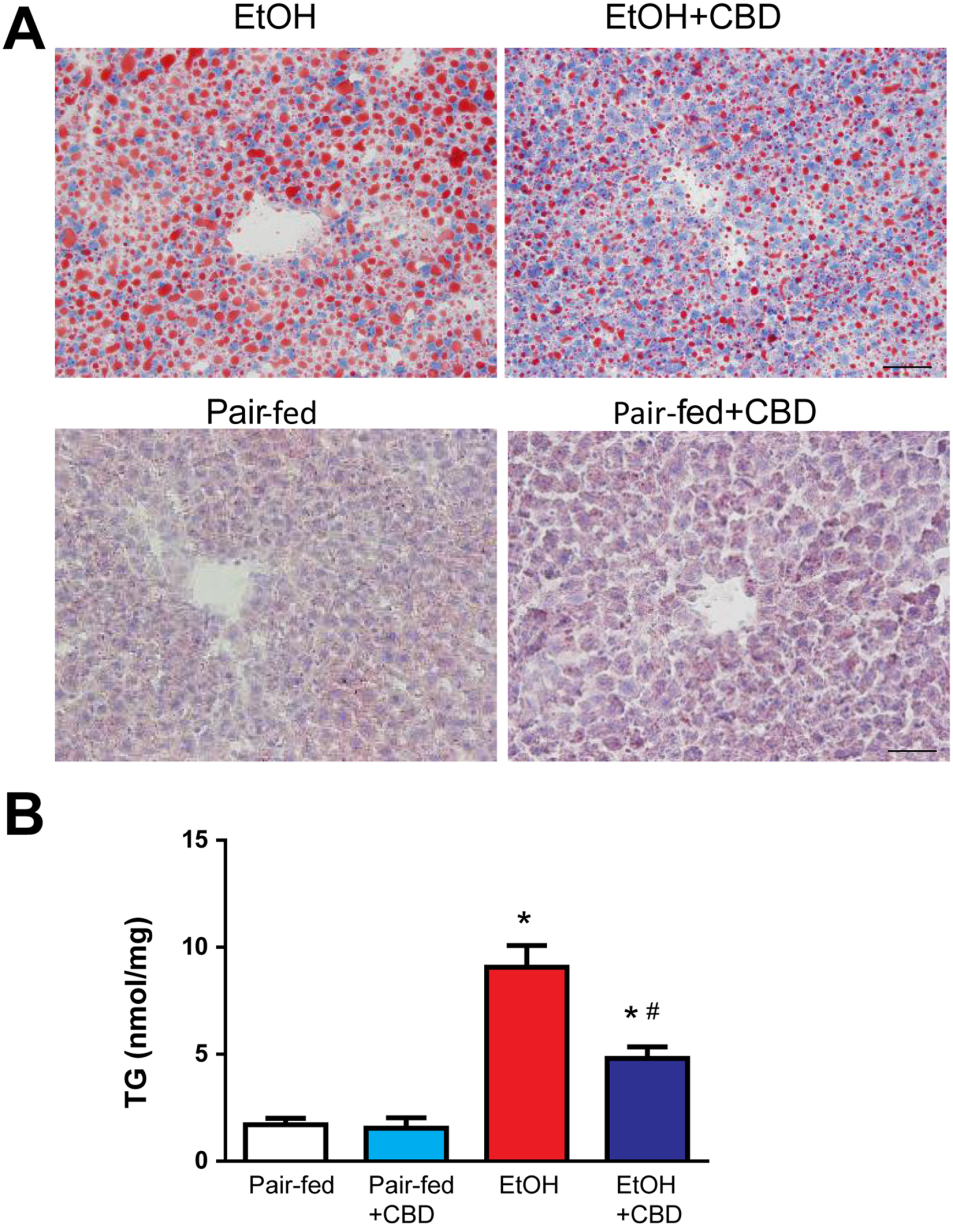
Effect of cannabidiol treatment on chronic-binge ethanol-induced liver steatosis. (**A**) Representative Oil red O staining of liver sections and (**B**) TG, liver triglyceride content from indicated groups. Values represent means ± SEM (n = 4–7). *P < 0.05 vs. Pair-fed group, ^#^ *P* < 0.05 vs. EtOH group determined by One-way ANOVA, followed by Tukey’s post-hoc test.

Chronic-plus-binge ethanol feeding induced significant liver injury as shown by H&E staining (Fig. 1A) and by elevated serum transaminases ALT and AST (Fig. 1B). Marked alcohol-induced hepatic lipid/triglyceride accumulation was also observed, indicated by Oil Red O staining (Fig. 2A) and elevated liver triglyceride content (Fig. 2B). All these pathological changes were markedly attenuated by CBD treatment (Figs 1 and 2). CBD treated did not cause any changes in pair-fed groups.

### Cannabidiol modulates genes and proteins involved in metabolism and liver steatosis

**Figure 3:**
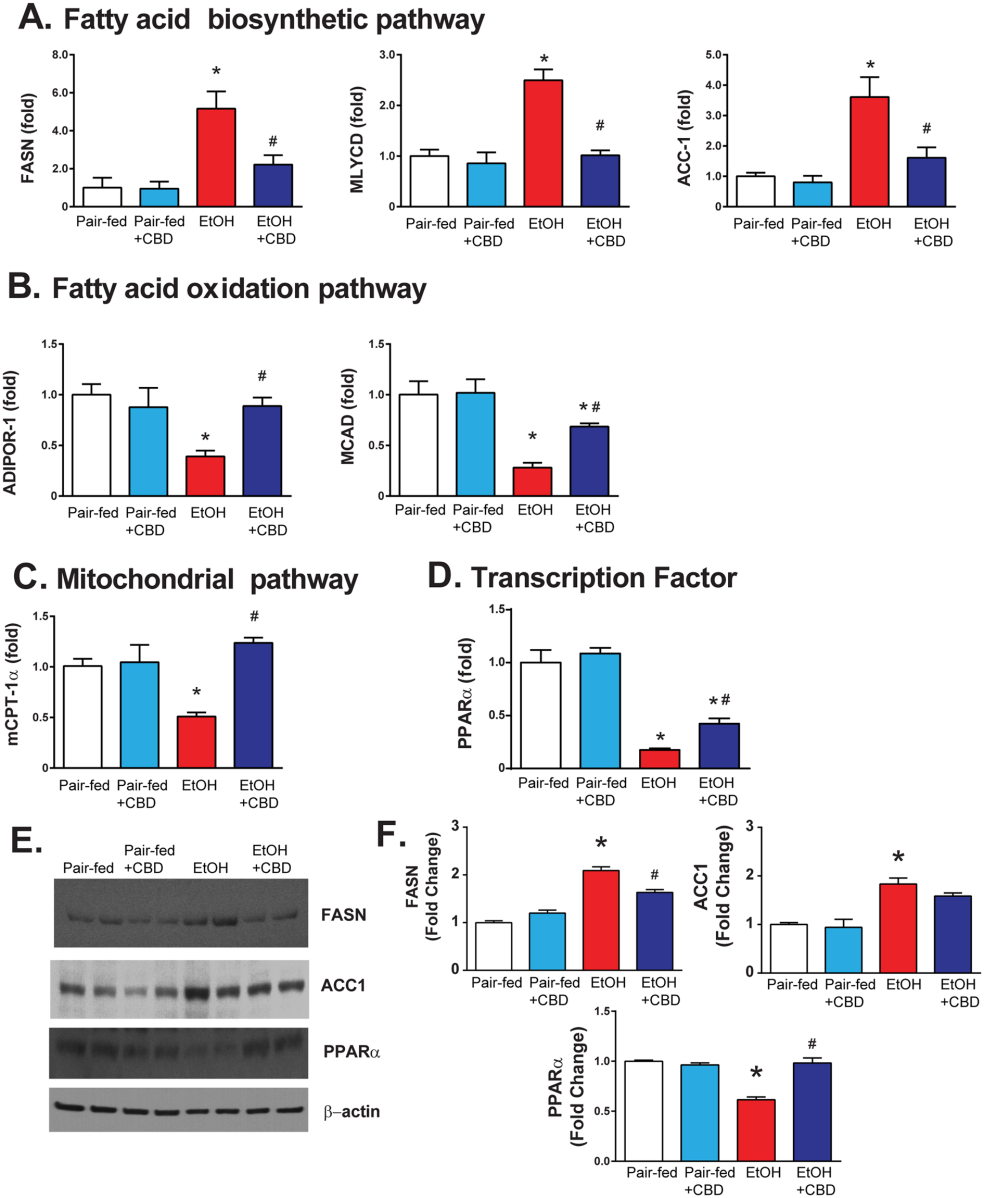
Effect of cannabidiol treatment on chronic-binge ethanol-induced dysregulation of metabolic genes in the liver. Realtime PCR analyses of genes involved in liver fatty acid biosynthetic (FASN, MLYCD and ACC-1) (**A**) and oxidation pathways (ADIPOR-1 and MCAD) (**B**), mitochondrial pathway (mCPT-1) (**C**) and transcription factor PPAR α (**D**). Values represent means ± SEM (n = 4–7). *P < 0.05 vs. Pair-fed group, ^#^ *P* < 0.05 vs. EtOH group determined by One-way ANOVA, followed by Tukey’s post-hoc test. (**E**) Western blot analyses of FASN, ACC1, PPARα with loading control β-actin. (**F**) Quantification of western blot data for FASN, ACC1 and PPARα. Values represent means ± SEM (n = 4/group). *P < 0.05 vs. Pair-fed group, ^#^ *P* < 0.05 vs. EtOH group determined by One-way ANOVA, followed by Tukey’s post-hoc test. The full-length blots are included in the Supplemental information file.

Because dysregulated metabolism plays an important role in alcohol-induced liver steatosis we examined a series of metabolic genes involved in fatty acid biosynthetic, oxidation and mitochondrial pathways (Fig. 3A–D). We found that alcohol enhanced hepatic expression of several genes involved in fatty acid biosynthesis (Fatty Acid Synthase (FASN), malonyl-CoA decarboxylase (Mlycd), and acetyl-Coenzyme A carboxylase alpha (ACC1)), while decreasing gene expressions involved in fatty acid oxidation (adiponectin receptor 1 (Adipor1) and medium-chain acyl-CoA dehydrogenase (MCAD); (Fig. 3AB). All these effects were attenuated by CBD treatment (Fig. 3AB). Alcohol feeding decreased hepatic carnitine palmitoyltransferase 1α (mCPT1α) and transcription factor peroxisome proliferator-activated receptor α (PPARα) mRNA expression levels (Fig. 3C,D). Alcohol also increased the protein expressions of FASN and ACC1 and decreased PPARα (Fig. 3E,F). These alcohol-induced effects were ameliorated by CBD treatment (Fig. 3C–F). CBD treatment had no effect on the above mentioned variables in pair-fed mice (Fig. 3A–D).

### Cannabidiol attenuates alcohol-induced neutrophil accumulation in the liver

**Figure 4:**
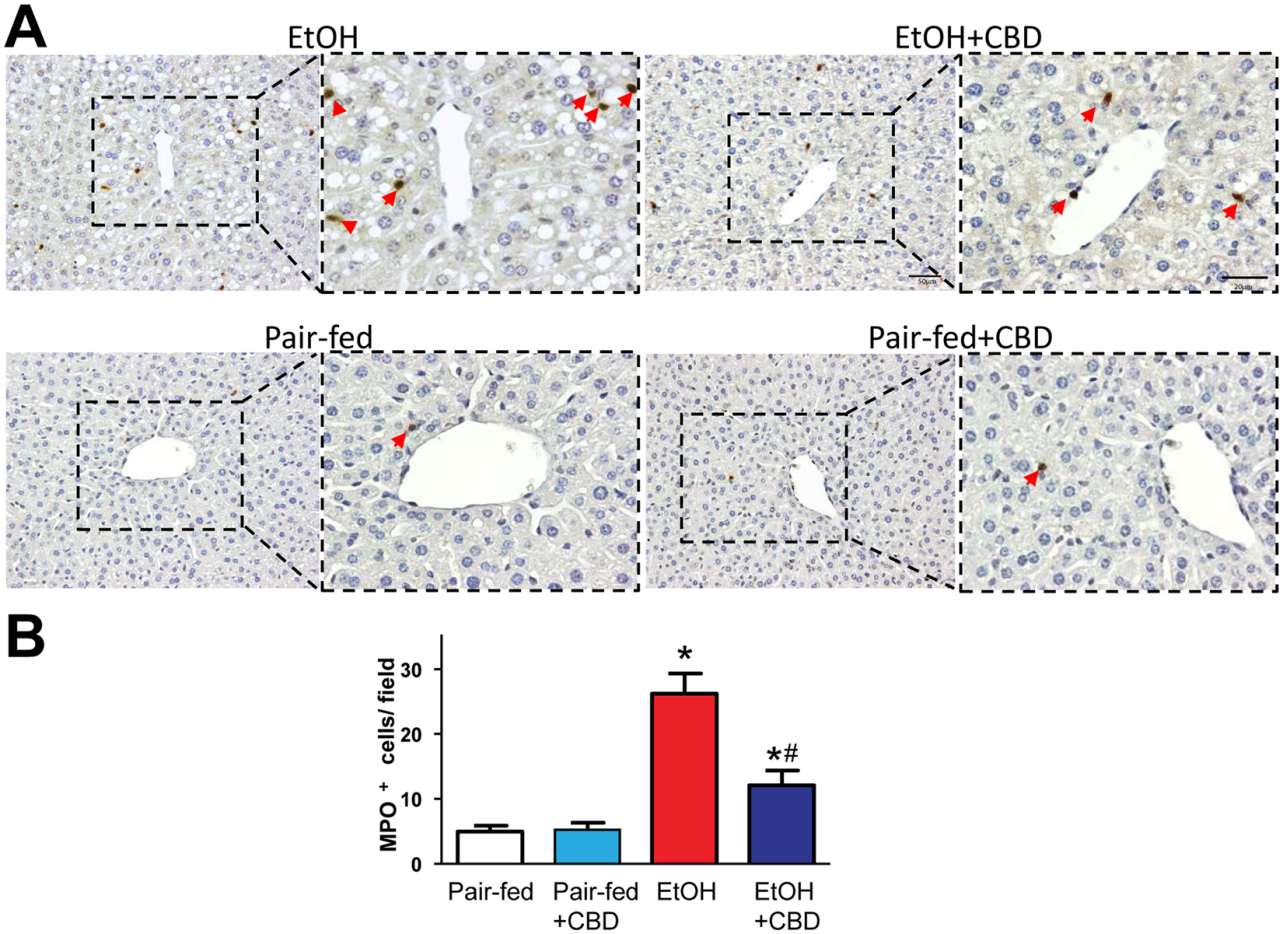
Effects of cannabidiol treatment on alcohol-induced liver neutrophil accumulation. (**A**) Representative liver showing myeloperoxidase (MPO) positive infiltrating neutrophils (original magnification, X200) and quantification (**B**). Arrows indicate MPO positive cells. Values represent means ± SEM. *P < 0.05 vs. Pair-fed group, ^#^ *P* < 0.05 vs. EtOH group determined by One-way ANOVA, followed by Tukey’s post-hoc test.

Chronic-plus-binge ethanol feeding induced significant hepatic neutrophil accumulation indicated by increased myeloperosidase (MPO) positive cells in histological sections, which was attenuated by CBD treatment (Fig. 4A,B).

### Cannabidiol attenuates oxidative burst in mouse and human neutrophils independent from cannabinoid 2 receptors

**Figure 5:**
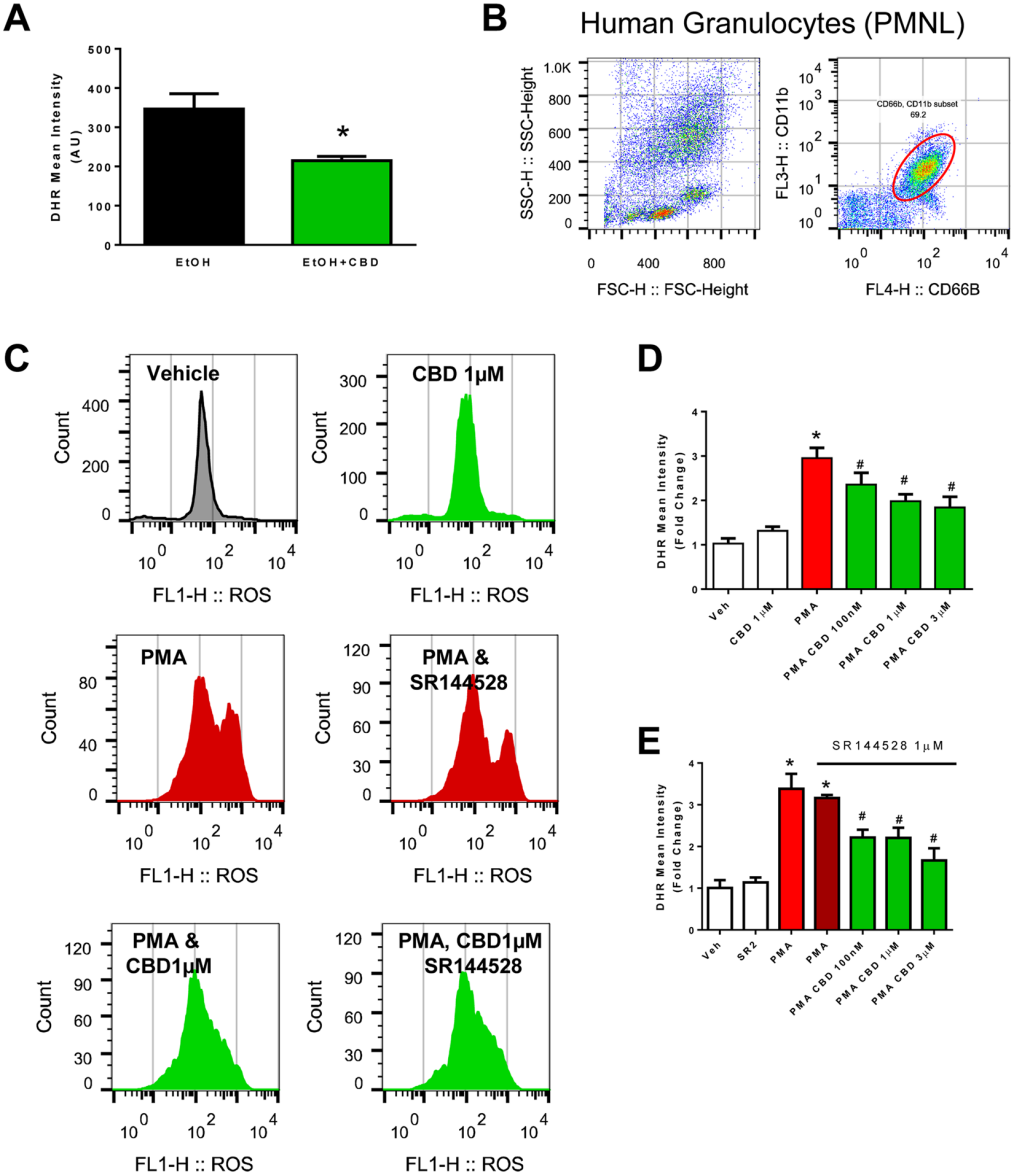
Effect of cannabidiol treatment on the hepatic neutrophil function in mice and in human neutrophils. (**A**) Neutrophils were isolated from livers of mice after chronic binge alcohol diet and incubated with PMA *in vitro*. The production of ROS was then measured (DHR mean intensity). Values represent means ± SEM (n = 3/group). *P < 0.05 vs. EtOH group determined by Student’s t-test. (**B**) Representative dot blot analyses of human granulocytes from blood using forward and side scatter followed by CD11b and CD66b staining. Double positive cells were gated for ROS measurement. (**C**) Representative histogram of ROS intensity (FL1) of human neutrophils treated with CBD (1 µM) and/or CB_2_ antagonist SR144528 (1 µM) followed by PMA treatment (100 µg/ml) *in vitro*. (**D,E**) Quantitative analyses of DHR mean intensity expressed as fold change (compared to vehicle). Values represent means ± SEM (n = 4/group). *P < 0.05 vs. vehicle group, ^#^ *P* < 0.05 vs. PMA group determined by One-way ANOVA, followed by Tukey’s post-hoc test.

CBD treatment of mice markedly attenuated the oxidative burst induced by PMA in neutrophil isolated from livers of ethanol-fed mice (Fig. 5A).

We also investigated the effects of CBD on PMA-induced oxidative burst in human neutrophils. CBD attenuated the PMA-induced neutrophil oxidative burst in concentration-dependent fashion (Fig. 5D) in human neutrophils. These beneficial effects of CBD could not be prevented by cannabinoid 2 receptor (CB_2_) antagonist SR144528 (Fig. 5C–E), indicating that this effect is independent from CB_2_.

### Cannabidiol attenuates liver oxidative/nitrative stress

**Figure 6:**
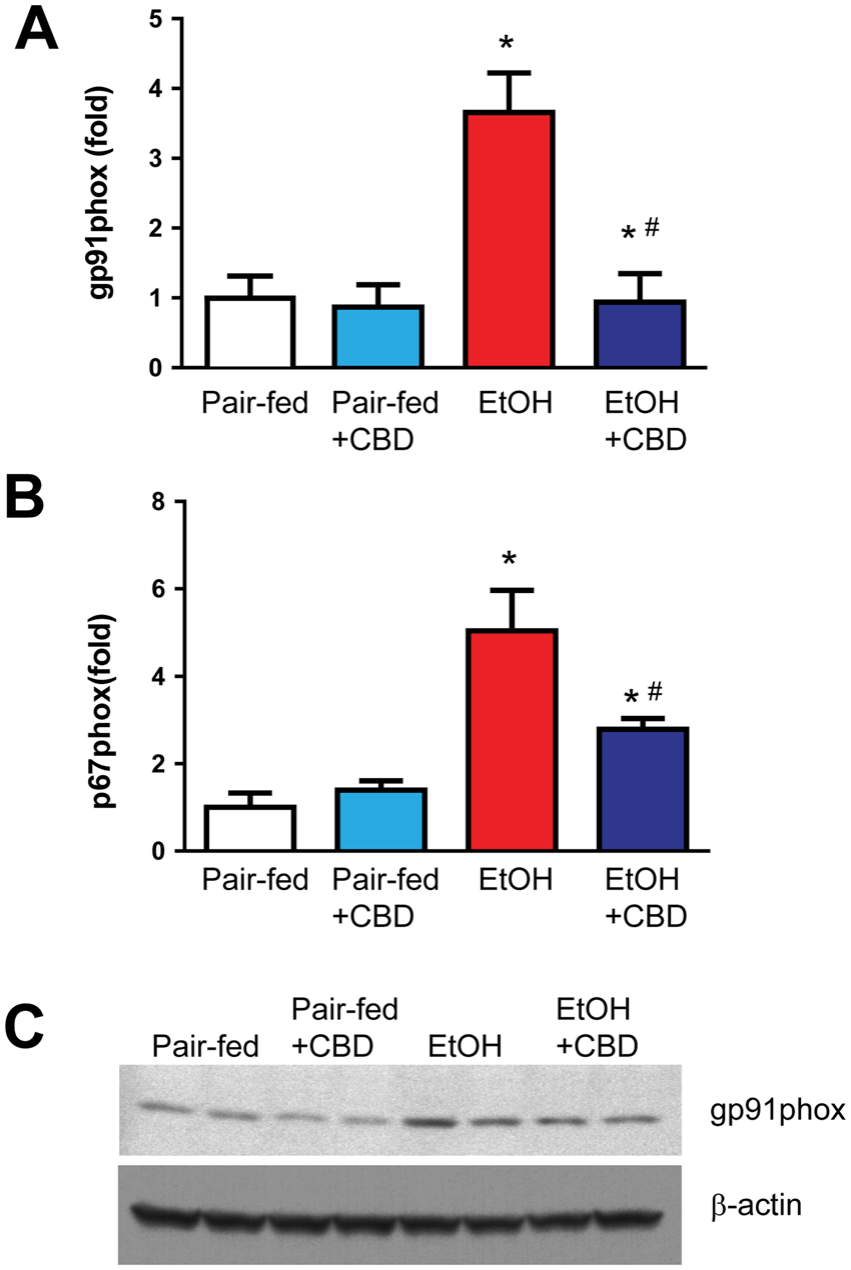
Effects of cannabidiol on alcohol-induced NAD(P)H oxidase isoforms mRNA and protein expression in the liver. Levels of NAD(P)H oxidase isoform NOX2/gp91phox (**A**) and p67phox (**B**) mRNA were analyzed by real-time PCR. The results are expressed as fold increases relative to the pair-fed group. Values represent means ± SEM (n = 5–7). *P < 0.05 vs. Pair-fed group, ^#^ *P* < 0.05 vs. EtOH group determined by One-way ANOVA, followed by Tukey’s post-hoc test. (**C**) Western blot analyses of gp91phox with loading control β-actin. The full-length blots are included in the Supplemental information file.

**Figure 7:**
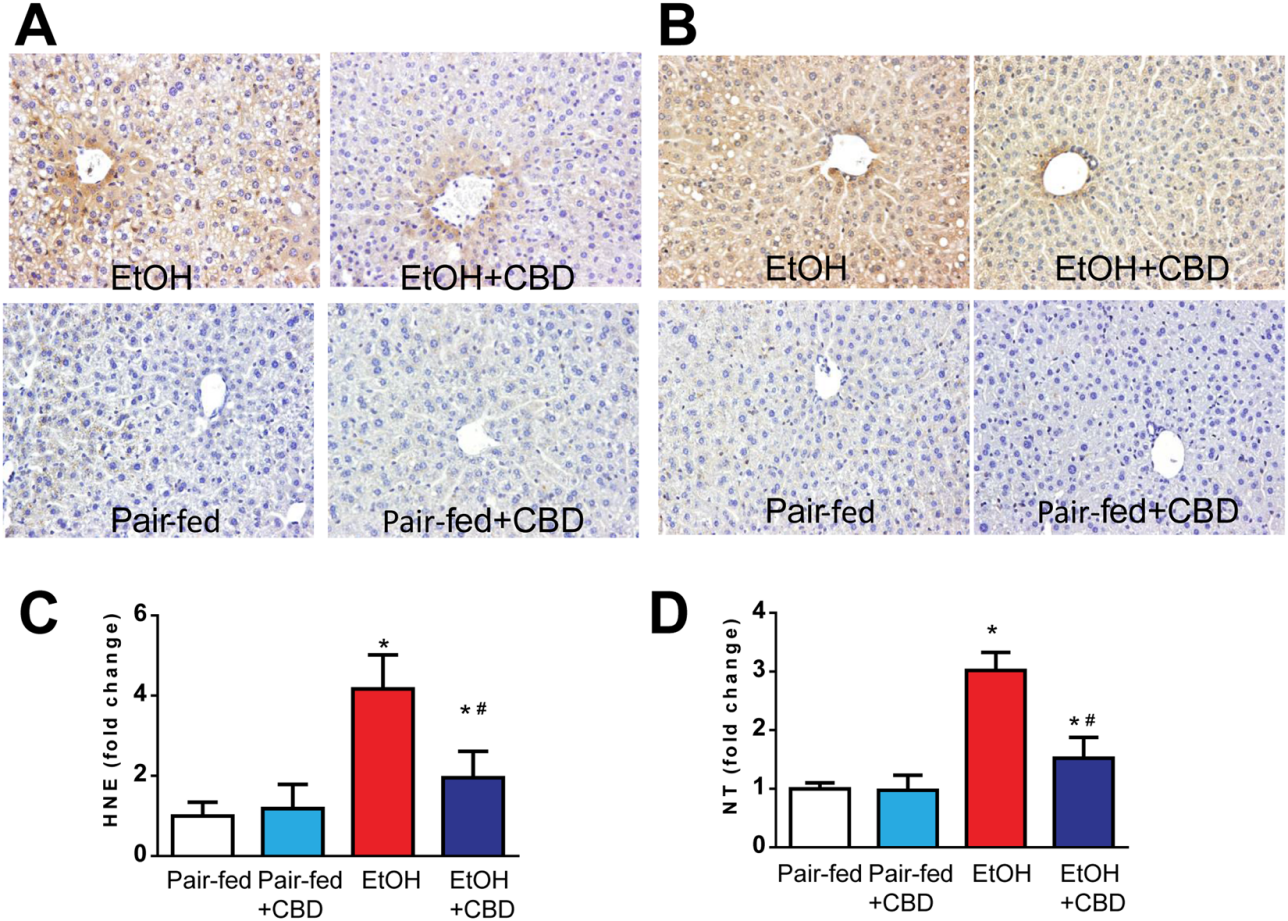
Effects of cannabidiol on ethanol-induced liver oxidative/nitrative stress. (**A,B**) Representative sections from each group with MDA and NT staining (markers of lipid peroxidation and protein nitration, respectively) at 200X magnification. Brown depicts increases of MDA and NT staining intensities in EtOH-fed groups. Panels (C) and (D) present quantification of HNE and NT ELISA respectively. Values represent means ± SEM (n = 5–7). *P < 0.05 vs. Pair-fed group, ^#^ *P* < 0.05 vs. EtOH group determined by One-way ANOVA, followed by Tukey’s post-hoc test.

Because the alcohol-induced liver injury is known to be associated with increased oxidative and nitrative stress we also examined the effect of CBD treatment on alcohol induced oxidative/nitrative stress markers. We found that alcohol feeding markedly enhanced the hepatic mRNA expression of reactive oxygen species (ROS) generating NADPH oxidase 2 (NOX2) isoforms gp91phox and p67phox (Fig. 6A,B), increased protein expression of gp91phox (Fig. 6C), malondialdehyde (MDA, marker of lipid peroxidation) and 3-nitrotyrosine (NT, marker of nitrative stress) immunostaining (Fig. 7AB) and 4-hydroxynonenal (HNE, marker of lipid peroxidation) and NT content (measured by ELISA; Fig. 7CD). CBD treatment significantly attenuated the alcohol diet-induced oxidative and nitrative stress in the liver (Figs 6 and 7). CBD treatment did not affect ROS in pair-fed groups.

### Cannabidiol attenuates chronic-binge ethanol-induced liver inflammation

**Figure 8:**
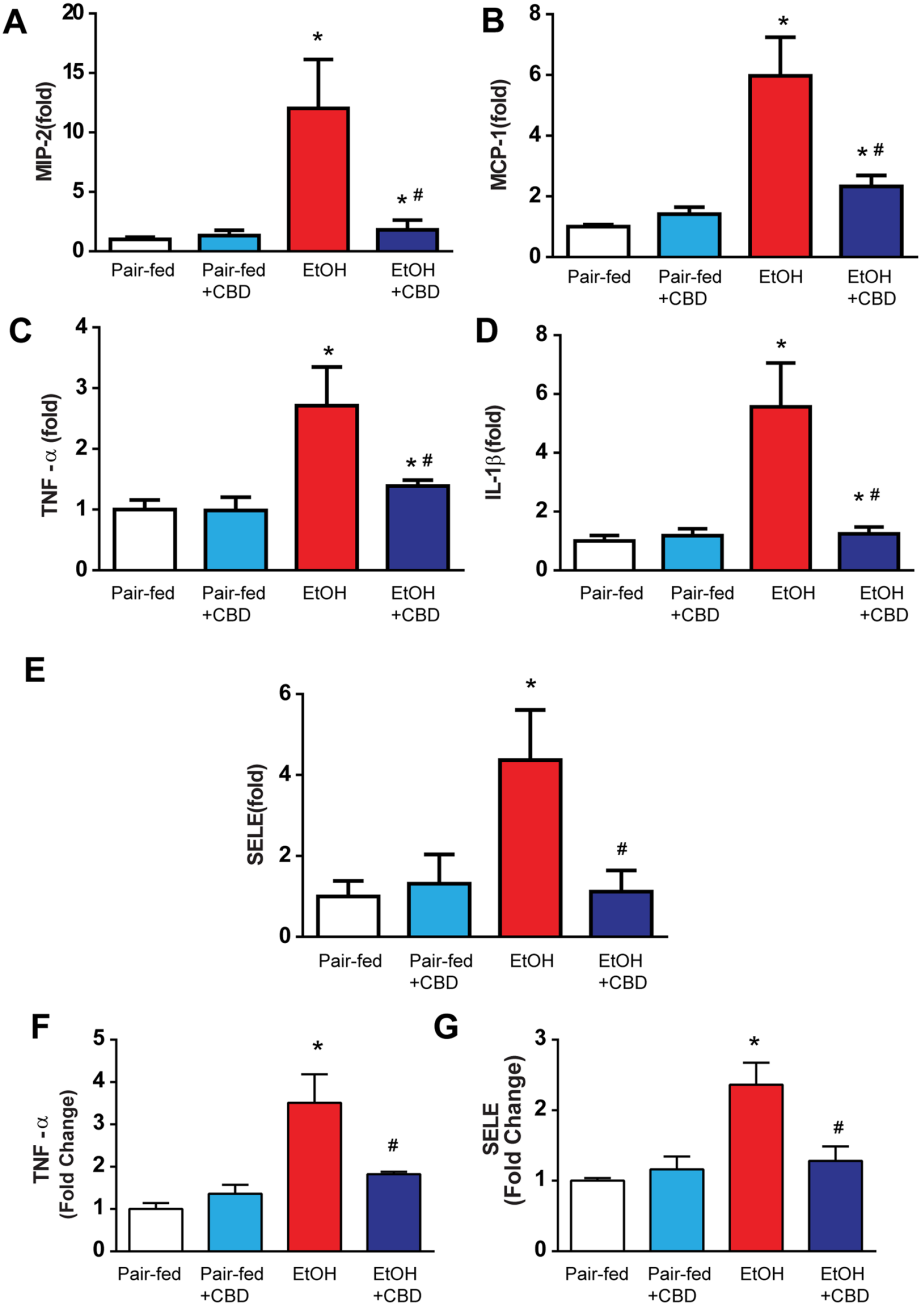
Effect of cannabidiol on ethanol-induced liver inflammation. Hepatic mRNA levels of MIP-2 (**A**), MCP-1 (**B**), TNF-α (**C**), IL-1β (**D**), and adhesion molecules E selectin (**E**) were analyzed by real-time PCR. The results are expressed as fold increases relative to the pair-fed groups. Values represent means ± SEM (n = 5–7). *P < 0.05 vs. Pair-fed group, ^#^ *P* < 0.05 vs. EtOH group determined by One-way ANOVA, followed by Tukey’s post-hoc test. (**F**) Quantitative ELISA of cytokine TNF-α (**F**) and E selectin (**G**) in liver tissue lysates. Values represent means ± SEM (n = 4/group). *P < 0.05 vs. Pair-fed group, ^#^ *P* < 0.05 vs. EtOH group determined by One-way ANOVA, followed by Tukey’s post-hoc test.

To understand the mechanism of the beneficial effects of CBD on alcohol-induced neutrophil accumulation in the liver, we studied the effect of CBD on alcohol induced pro-inflammatory chemokines, cytokines and adhesion molecules. Chronic CBD treatment markedly decreased alcohol diet-induced increased hepatic mRNA expressions of pro-inflammatory chemokines (macrophage inflammatory protein 2-alpha (MIP-2)/chemokine (C-X-C motif) ligand 2 (CXCL2), monocyte chemotactic protein 1 (MCP-1/CCL2)), cytokines (tumor necrosis factor alpha (TNF-α) and interleukin 1 beta (IL1-β)), and adhesion molecule selectin E (SELE)(Fig. 8A–E). It also attenuated the hepatic TNFα and SELE levels measured by ELISA. CBD had no significant effect on these variables in pair-fed mice.

## Discussion

Chronic alcohol consumption is a major cause of alcoholic liver disease characterized by metabolic dysregulation, enhanced oxidative stress, inflammation and steatosis in the liver, which may eventually culminate in cirrhosis or hepatocellular carcinoma in susceptible subjects^1,2^. Despite significant advances in our understanding of the pathology of alcoholic liver disease the therapeutical options are still very limited.

The recently FDA approved for exploratory trials non-psychoactive constituent of Cannabis sativa, cannabidiol (CBD), has been reported to exert antioxidant, cytoprotective and anti-inflammatory effects independently of cannabinoid 1 and 2 receptors in preclinical disease models.

In the present study, we describe that CBD treatment improves chronic-plus-binge-alcohol-induced hepatocellular liver injury in mice by attenuating oxidative and nitrative stress, expression of adhesion molecule E-selectin, neutrophil infiltration and neutrophil-mediated oxidative injury and inflammation. We also show that CBD attenuates alcohol-induced liver steatosis and dysregulation of numerous key genes of fatty acid biosynthetic and oxidation pathways, mitochondrial pathways, and transcription factor PPARα, implicated in development of alcohol-induced steatohepatitis. Furthermore, we demonstrate that CBD attenuates the oxidative burst in human neutrophils independently from anti-inflammatory cannabinoid 2 receptor.

Chronic-plus-binge alcohol leads to hepatocyte necrosis in the liver, causing proinflammatory response. It is known that neutrophils are linked to alcoholic hepatitis^22,33^. We also found increased neutrophil recruitment in livers and enhanced expressions of ROS generating NAD(P)H enzyme NOX2 isoforms, coupled with enhanced oxidative and nitrative stress in our model, which were all attenuated by CBD treatment. CBD treatment also attenuated the response of neutrophils isolated from alcoholic livers to generate ROS upon stimulation. Consistent with its previously described antioxidant effect^7,11^, CBD treatment also decreased the neutrophil burst in human neutrophils *in vitro* independent from anti-inflammatory CB_2_ receptor.

Numerous recent studies described potent anti-inflammatory effects of CBD reaching far beyond its initially reported antioxidant properties in models of colitis, ischemic-reperfusion injury, diabetic complications, autoimmune arthritis, hepatitis, myocarditis and neuroinflammation^6,9,11,12,16,34^. One of the most characteristic effects of CBD in these preclinical models of inflammation is the attenuation of the inflammatory cell infiltration and pro-inflammatory response^6^. In agreement with these reports in our chronic plus binge alcohol induced liver injury model, CBD suppressed the alcohol induced pro-inflammatory cytokines/chemokines (TNFα, IL-1β, MIP2 and MCP-1) expressions/levels in the liver.

Kupffer cell polarization (M1-M2 phenotype) has been importantly implicated in the initiation of alcoholic liver diseases^35^. In the current study CBD treatment significantly reduced expression of M1-related genes (TNFα and MCP-1) suggesting that M1 polarization could contribute to the anti-inflammatory effects of CBD, which should be explored in the future studies.

E-selectin/CD62/or endothelial-leukocyte adhesion molecule 1 is a key adhesion factor expressed in activated endothelium, which is involved in recruitment of leukocytes, particularly neutrophils during tissue injury^36^. An important role of E selectin in chronic plus binge alcohol-induced liver injury has recently been suggested^25^. Interestingly, CBD treatment attenuated the alcohol-induced hepatic E-selectin expression, consistently with its anti-inflammatory effects in activated endothelium^37^.

Most intriguingly CBD also attenuated the alcohol-induced metabolic dysregulation and steatosis in the liver. These results suggests that CBD may exert direct beneficial effects on lipid metabolism independent from its antioxidant and anti-inflammatory effects, which is consistent with a recent report demonstrating attenuation of lipid accumulation in hepatocytes and adipocytes by CBD *in vitro*, as well as in obese mice *in vivo* ^21^.

In conclusion, we demonstrate that CBD treatment significantly attenuates liver injury induced by chronic plus binge alcohol in a mouse model and oxidative burst in human neutrophils. CBD ameliorates alcohol-induced liver injury by attenuating inflammatory response involving E-selectin expression and neutrophil recruitment, and consequent oxidative/nitrative stress, in addition to attenuation of the alcohol-induced hepatic metabolic dysregulation and steatosis. These beneficial effects, coupled with the proven safety of CBD in human clinical trials and its current orphan drug approval by FDA for various indications suggest that it may have therapeutic potential in liver disease associated with inflammation, oxidative stress, metabolic dysregulation and steatosis.

## Electronic supplementary material

Supplementary Data
