# Supplementary material for: Cannabidiol attenuates alcohol-induced liver steatosis, metabolic dysregulation, inflammation and neutrophil-mediated injury
Source: Sci Rep. 2017 Sep 21;7:12064. doi: 10.1038/s41598-017-10924-8 (PMC5608708; doi:10.1038/s41598-017-10924-8)
Supplement: Supplementary file 1 — Supplementary Data [file 41598_2017_10924_MOESM1_ESM.pdf]

Scientific Reports R1 May 16, 2017

Cannabidiol attenuates alcohol-induced liver steatosis, metabolic dysregulation, inflammation and neutrophil-mediated injury

Yuping Wang[1,2], Partha Mukhopadhyay[1], Zongxian Cao[1], Hua Wang[3], Dechun Feng[3], György Haskó[4], Raphael Mechoulam[5], Bin Gao[3] and Pal Pacher[1*]

[1] Laboratory of Cardiovascular Physiology and Tissue Injury, National Institute on Alcohol Abuse and Alcoholism, National Institutes of Health, Bethesda, MD, USA.

[2]Department of Clinical Microbiology and Immunology, Affiliated Hospital of Guiyang Medical University, Guiyang, Guizhou Province, China

[3]Laboratory of Liver Diseases, National Institute on Alcohol Abuse and Alcoholism, National Institutes of Health, Bethesda, MD, USA

[4]Department of Surgery and Center for Immunity and Inflammation, Rutgers New Jersey Medical School, Newark, New Jersey, USA

[5]Institute for Drug Research, Medical Faculty, Hebrew University, Jerusalem, 91120, Israel

Correspondence to:

Pal Pacher, M.D., Ph.D., F.A.H.A., F.A.C.C.
Chief, Laboratory of Cardiovascular Physiology and Tissue Injury, NIAAA, NIH
e-mail: pacher@mail.nih.gov
phone: (301)443-4830

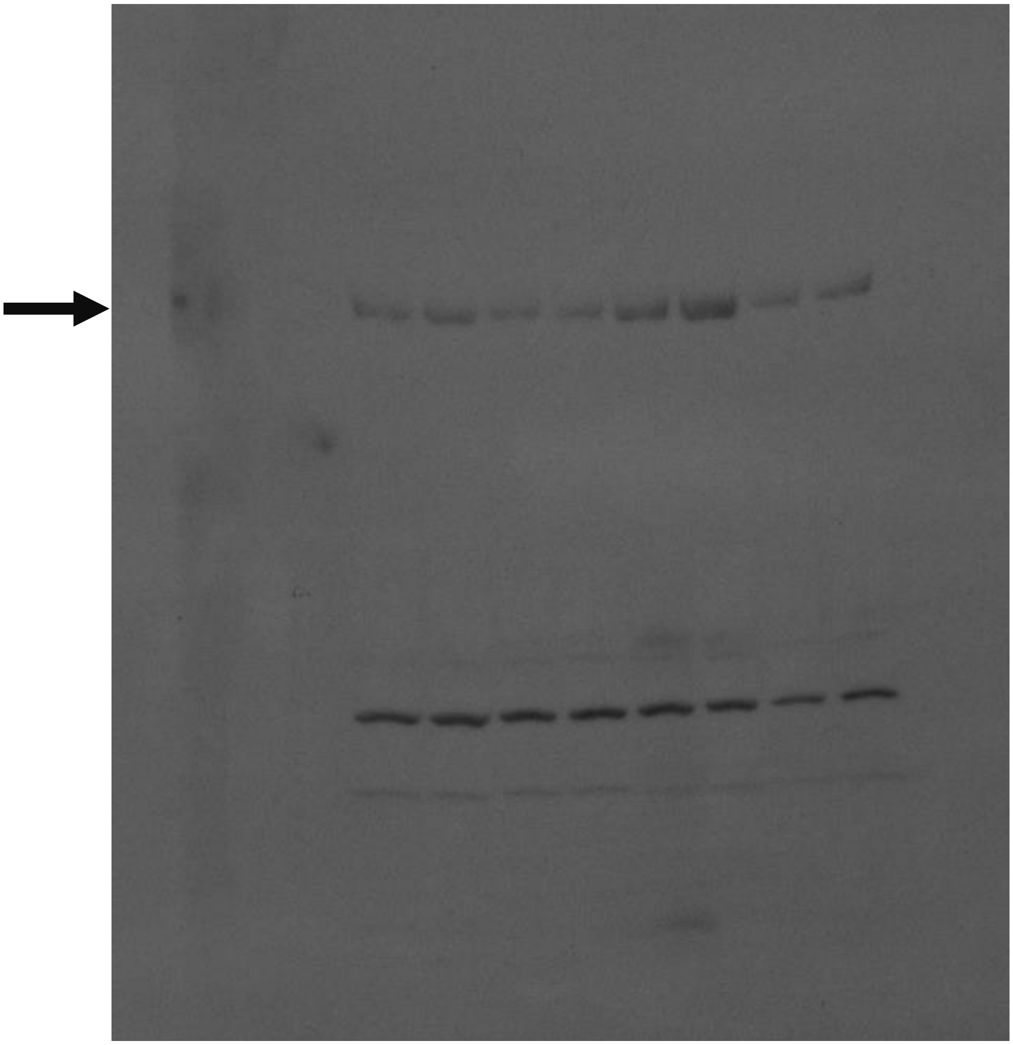

Figure S1: Representative original blot FASN as shown in figure 3E.

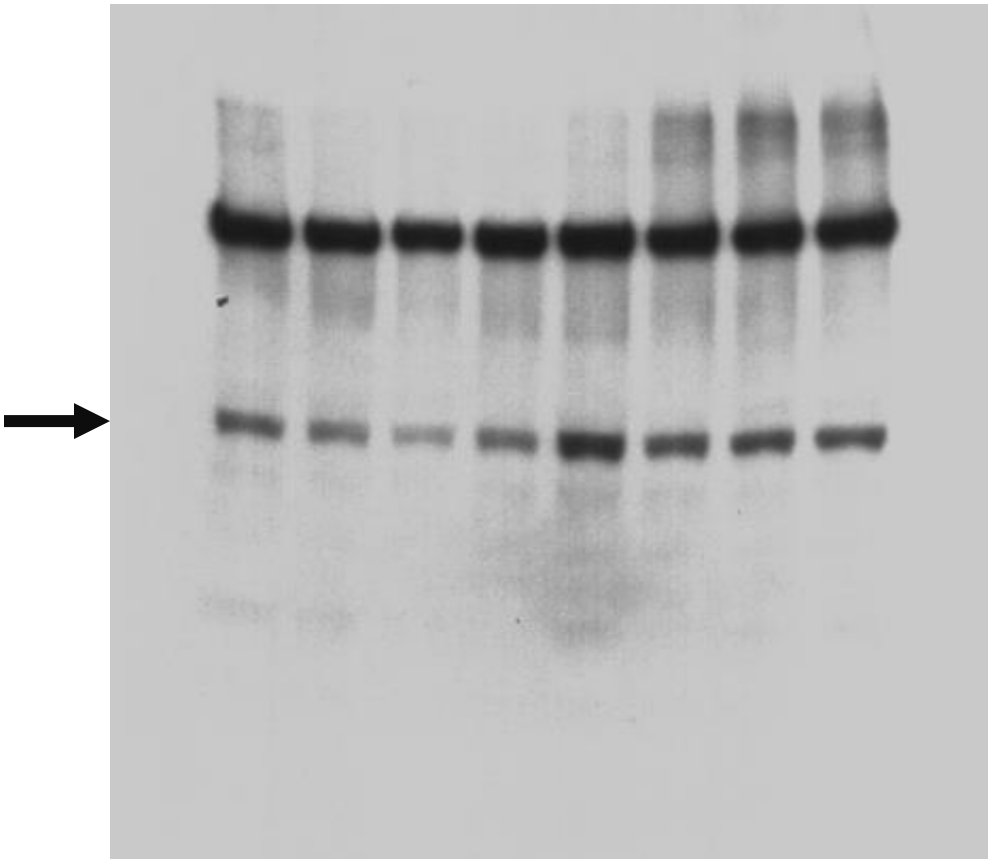

Figure S2: Representative original blot of ACC1 as shown in figure 3E.

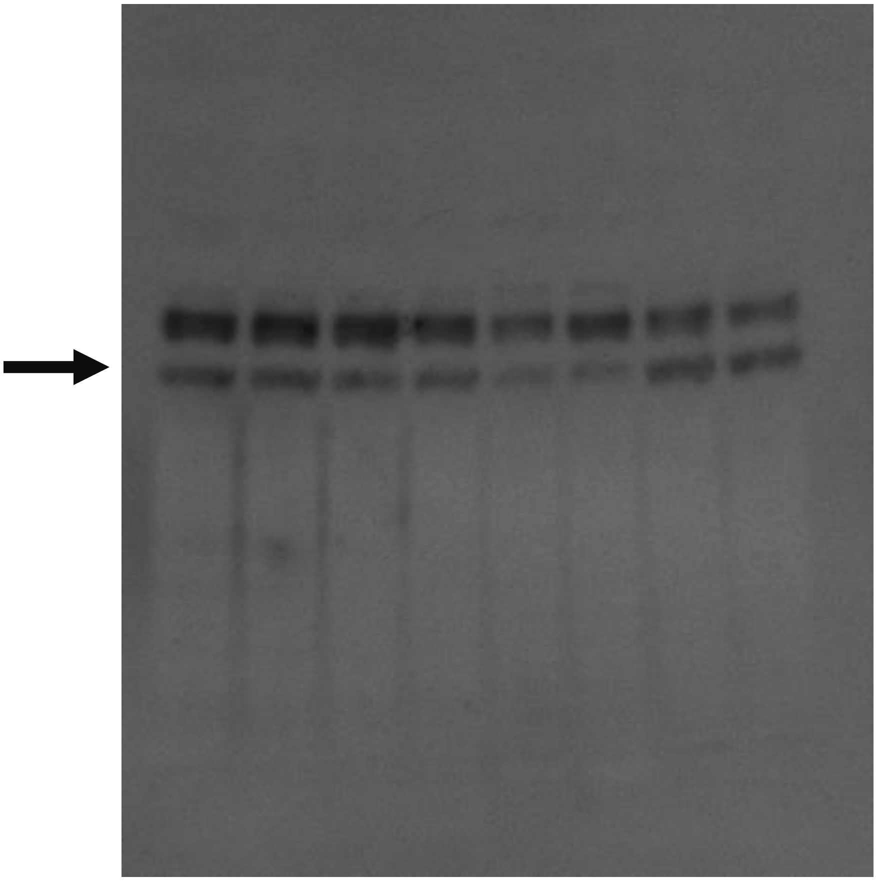

Figure S3: Representative original blot of PPARα as shown in figure 3E.

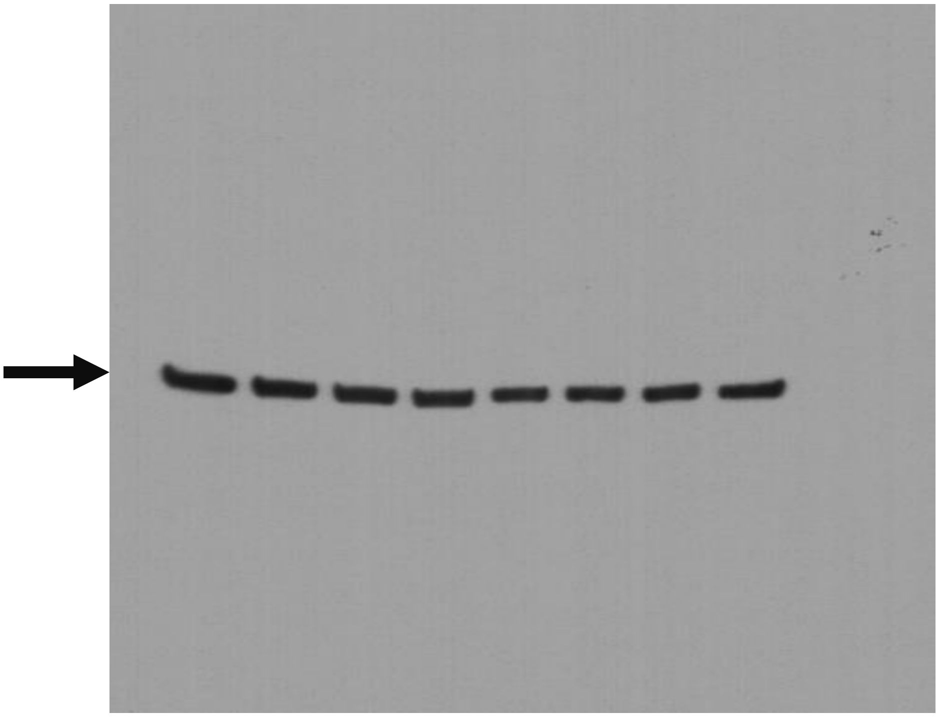

Figure S4: Representative original blot of β-actin as shown in figure 3E.

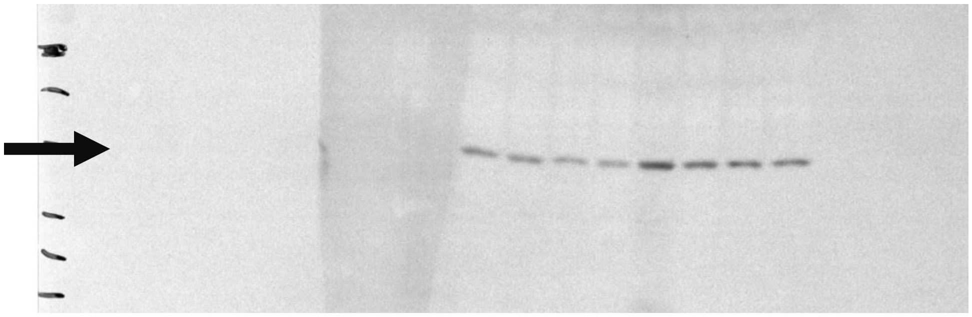

Figure S5: Representative original blot of gp91phox as shown in figure 6C

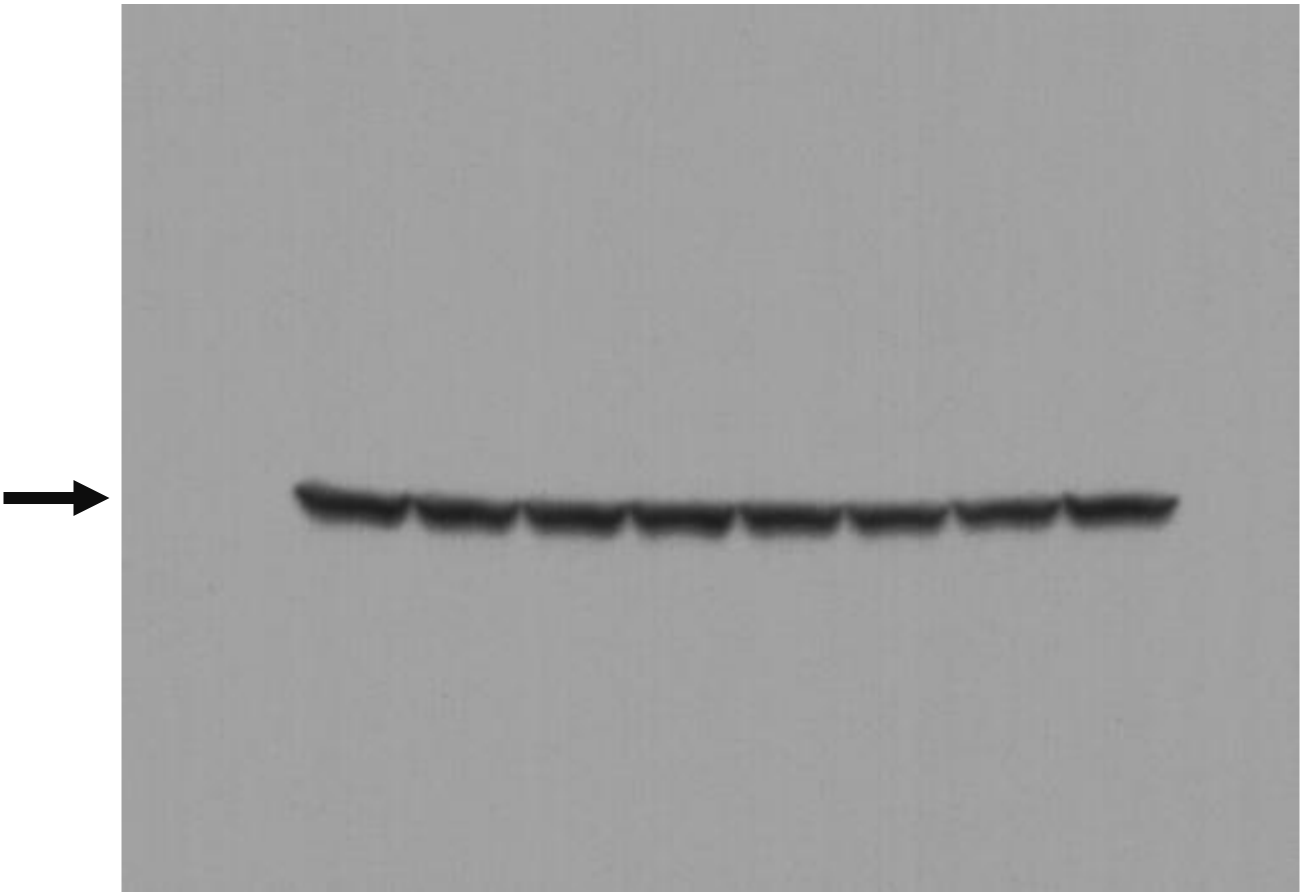

Figure S6: Representative original blot of β-actin as shown in figure 6C
